# Surveillance for endometrial cancer with transvaginal ultrasonography of breast cancer patients under tamoxifen treatment

**DOI:** 10.1038/sj.bjc.6600894

**Published:** 2003-04-15

**Authors:** S Ciatto, S Cecchini, G Gervasi, A Landini, M Zappa, E Crocetti

**Affiliations:** 1 Centro per lo Studio e la Prevenzione Oncologica, Viale A. Volta 171, 50131 Firenze, Italy

**Keywords:** endometrial cancer, diagnosis, endometrial thickness, transvaginal ultrasonography, tamoxifen

## Abstract

The association of endometrial thickness with the risk of developing endometrial cancer (EC) within 2 years was investigated in a consecutive cohort of 1205 breast cancer patients under tamoxifen treatment, undergoing transvaginal ultrasonography (TVUS) for follow-up purpose (asymptomatic, 1068) or for abnormal uterine bleeding (AUB, 137). Linkage with tumour registry allowed for the follow-up of 3184.3 person-years. According to underlying incidence, 1.85 EC cases were expected in the study cohort while 12 were observed (observed/expected ratio=6.49, 95% CI 3.35–11.33; asymptomatic=4.09, 95% CI 1.65–8.43, symptomatic=35.71, 95% CI 11.59–83.34). No EC was observed with thickness (half layer) <3 mm. Raising this threshold increased specificity with a substantial loss of sensitivity (⩾3, ⩾4, ⩾6, ⩾9 mm; spec.=25.8, 44.5, 76.1, 91.5%, sens.=100, 91.6, 75.0, 66.6%). The presence of AUB was rather specific (88.94%) but poorly sensitive (41.67%). A combination of AUB presence/absence and thickness allowed the best accuracy (AUB + thickness ⩾3, ⩾4 or ⩾5; sens.=100, 81.6 or 91.6%; spec.=22.8, 40.4, or 56.7%). Breast cancer patients under tamoxifen might be selected for further invasive assessment on the basis of AUB and endometrial thickness assessed at TVUS.

Several studies report an increase of endometrial cancer (EC) risk in breast cancer patients after long-term tamoxifen treatment (Fornander *et al*, 1989; Fisher *et al*, 1994, Cecchini *et al*, 1998); such a finding is only partially explained by the association between EC and breast cancer (Teppo *et al*, 1985; Crocetti *et al*, 2001), and tamoxifen is most likely responsible for the large part of such increased risk. For this reason, special surveillance for EC has been suggested in breast cancer patients under adjuvant tamoxifen treatment (Jordan and Morrow, 1994; Neven *et al*, 1994) and periodic examination by transvaginal ultrasonography (TVUS) is commonly performed (Oladipo, 1993; Kedar *et al*, 1994). Increased endometrial thickness measured at TVUS has been reported as an indicator of endometrial proliferation and EC risk (Karlsson *et al*, 1995; Weber *et al*, 1997) and has been suggested as a criterion to prompt invasive diagnostic assessment (hysteroscopy, dilatation and curettage) in postmenopausal asymptomatic or symptomatic (AUB=abnormal uterine bleeding) women (Goldstein *et al*, 1990; Osmers *et al*. 1990; Cecchini *et al*, 1996; Langer *et al*, 1997; Smith-Bindman *et al*, 1998).

At the Centro per lo Studio e la Prevenzione Oncologica (CSPO) of Florence, a large number of breast cancer patients under tamoxifen treatment are currently followed up and periodic TVUS examination has been commonly performed since many years (Cecchini *et al*, 1998). A retrospective study has been designed with the purpose of assessing whether endometrial thickening is associated with increased risk of EC and may be used as an indicator for invasive assessment.

## MATERIALS AND METHODS

Subjects eligible for the study were postmenopausal (amenorrhaea since 1 year at least) breast cancer patients under tamoxifen treatment since at least 1 year, referring to CSPO for TVUS as a periodic control (asymptomatic) or because of abnormal uterine bleeding (AUB, symptomatic) from November 1993 to December 1999. No standard interval following onset of tamoxifen therapy was adopted, and reliable data on total duration of treatment were not available. TVUS was carried out with an SS250 Toshiba sonograph using a dedicated transvaginal convex 7 MHz transducer. Data available for each case were: name, birth date, TVUS date, symptoms (none, AUB), endometrial thickness in millimetres (single layer, determined on the longitudinal scan at the point of maximum thickness), diagnostic report (negative, benign, suspicious for endometrial cancer).

Eligible cases were linked with the Tuscany Tumour Registry database (linkage by name and date of birth) to identify subjects with EC (ICDO=1820) incident after TVUS date until December 1999. Patterns (median time from TVUS) of EC occurrence in symptomatic and asymptomatic cases were compared. Statistical analysis was based on Student's *t*-test to compare means of unmatched samples and Pearson *χ*^2^ to compare medians. Standardised (European population) incidence rates were calculated according to person-years in the overall series and according to symptomatic status. The number of EC cases observed in the study cohort was compared to that expected according to cancer registry age-adjusted incidence rates in the general population, and observed/expected (O/E) ratio and its 95% confidence interval (95% CI) were calculated. Sensitivity, specificity and negative/positive predictive values for EC were determined for different cutoffs of endometrial thickness. Receiver operating characteristics (ROC) curve analysis were also applied for different endometrial thickness cutoffs. We evaluated the possible clinical use of endometrial thickness measured at TVUS as a choice criterion between surveillance and immediate invasive assessment (hysteroscopy, dilatation and curettage) for asymptomatic and symptomatic subjects.

## RESULTS

The study cohort consists of 1205 eligible subjects (age range 37–92 years, median 64) of whom 1.068 were asymptomatic and 137 were symptomatic (no age difference was evident according to age: data not reported). Subjects had been followed up for a total of 3184.3 person-years (asymptomatic=2949.7; symptomatic= 244.6). Linkage with tumour registry files identified incident EC in 12 subjects (asymptomatic=7, symptomatic=5). The main features of these cases are shown in Table 1

**Table 1:** Age and symptoms at TVUS, TVUS report, endometrial thickness (half layer) and TVUS−endometrial cancer diagnosis interval in 12 EC cases observed in the present study

**Case**	**Age**	**Symptoms**	**TVUS report**	**Thickness (mm)**	**TVUS−EC diagnosis interval**	**Histological grading**	**% Myometrial invasion**
1	64	AUB	Suspicious	9	13.80 (months)	Unknown	50%
2	59	AUB	Suspicious	10	0.10	G1	Intramucous
3	66	AUB	Suspicious	10	8.31	G2	20%
4	55	None	Suspicious	9	4.44	G1	Intramucous
5	71	AUB	Suspicious	9	3.84	G2	40%
6	74	AUB	Negative	3	15.54	G1	5%
7	57	None	Suspicious	5	3.61	G2	45%
8	62	None	Suspicious	10	2.00	G2	40%
9	50	None	Negative	4	0.95	G2	30%
10	67	AUB	Suspicious	6	10.68	G3	40%
11	75	None	Suspicious	9	3.19	G1	20%
12	75	AUB	Suspicious	10	12.42	G3	60%

. Endometrial cancer was diagnosed in the average 6.57 months after TVUS (range 0.10–15.54 months, s.d. 5.34): the interval between TVUS and EC diagnosis was significantly shorter for symptomatic (average 2.84 months, range 0.95–4.44 months, s.d. 1.37) as compared to asymptomatic cases (average 9.24 months, range 0.10–15.54 months, s.d. 5.57, *P*=0.03). Histological grading was available in 11 cases (G1=4, G2=5, G3=2). Myometrial invasion was observed in 10 of 12 cases. High-grade (G3) and extensive (>50%) myometrial invasion was observed in cases with AUB.

The standardised EC incidence rate was 15.29 × 1000 (symptomatic=84.08, asymptomatic=5.50). Based on incidence rates in the general population (cancer registry) and person-years, 1.85 EC cases were expected in the study cohort while 12 were observed with an O/E ratio of 6.49 (95% CI 3.35–11.33). Corresponding values for asymptomatic or symptomatic cases were 4.09 (95% CI 1.65–8.43) and 35.71 (95% CI 11.59–83.34), respectively.

Table 2

**Table 2:** Distribution of the study cohort according to presence of EC cancer, symptoms and endometrial thickness (half layer)

	**Total cases**		**Symptomatic**		**Asymptomatic**	
**Thickness (mm)**	**Cancer**	**Noncancer**	**Cancer**	**Noncancer**	**Cancer**	**Noncancer**
0	0	14	0	1	0	13
1	0	94	0	17	0	77
2	0	200	0	17	0	183
3	1	223	0	14	1	209
4	1	210	1	15	0	195
5	1	168	1	10	0	158
6	1	76	0	12	1	64
7	0	62	0	6	0	56
8	0	45	0	12	0	33
9	4	23	2	5	2	18
10	4	31	1	7	3	24
11	0	7	0	5	0	2
12	0	6	0	1	0	5
13	0	4	0	3	0	1
14	0	3	0	1	0	2
15	0	8	0	1	0	7
16	0	5	0	2	0	3
17	0	3	0	1	0	2
18	0	1	0	0	0	1
19	0	2	0	2	0	0
20	0	8	0	0	0	8
						
Total	12	1193	5	132	7	1061

shows the distribution of asymptomatic/symptomatic, cancer/noncancer cases by endometrial thickness. Sensitivity and specificity for EC occurring within 2 years from TVUS calculated for different cutoffs of endometrial thickness are shown in Table 3

**Table 3:** Number of EC delayed diagnoses and of unnecessary assessments, sensitivity, specificity and predictive values (PV) for different cutoffs of endometrial thickness (half layer) and for different protocols prompting immediate invasive diagnostic assessment for EC

**Invasive assessment if:**	**EC delayed diagnoses**	**Unnecessary assessments**	**Sens %**	**Spec %**	**PPV %**	**NPV %**
Thickness ⩾2	0	1085	100	9.05	1.09	100
Thickness ⩾3	0	885	100	25.82	1.34	100
Thickness ⩾4	1	662	91.67	44.51	1.66	99.81
Thickness ⩾5	2	452	83.33	62.11	2.16	99.63
Thickness ⩾6	3	284	75.00	76.19	3.07	99.67
Thickness ⩾7	4	208	66.67	82.56	3.70	99.60
Thickness ⩾8	4	146	66.67	87.76	5.19	99.62
Thickness ⩾9	4	101	66.67	91.53	7.34	99.64
AUB	7	132	41.67	88.94	3.65	99.34
Suspicious TVUS	2	438	83.33	63.29	2.23	99.74
AUB/thickness ⩾3	0	920	100	22.88	1.29	100
AUB/thickness ⩾4	1	711	91.67	40.40	1.52	99.79
AUB/thickness ⩾5	1	516	91.67	56.75	2.09	99.85
AUB/thickness ⩾6	1	358	91.67	69.99	2.02	99.88
AUB/thickness ⩾7	2	294	83.33	75.36	3.29	99.78
AUB/thickness ⩾8	2	238	83.33	80.05	4.03	99.79
AUB/thickness ⩾9	2	205	83.33	82.82	4.65	99.80
AUB/suspicious TVUS	1	476	91.67	60.10	2.26	99.86

**Figure 1:**
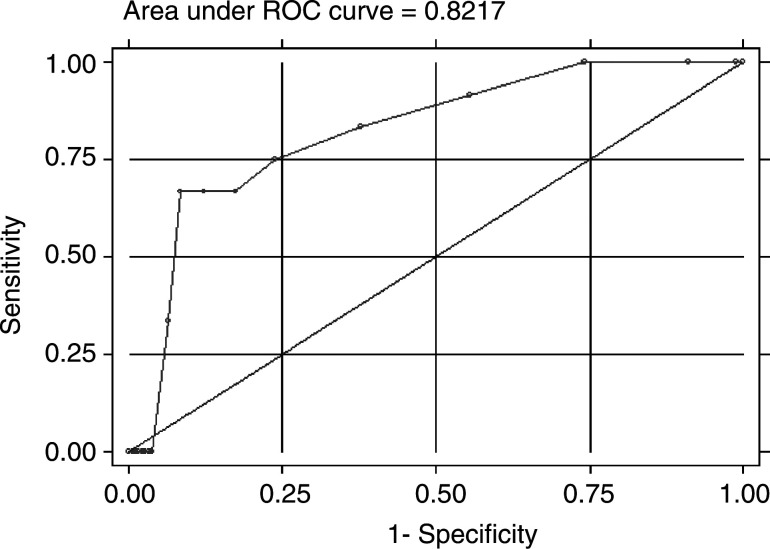
ROC curve of sensitivity and specificity for EC based on different cutoff levels (1–20 mm) of endometrial thickness (half thickness).

. A cutoff ⩾3 mm was associated with 100% sensitivity but with a relatively low specificity (25.82%). To increase specificity to >40 (cutoff ⩾4 mm), >75 (⩾6 mm) or >90% (⩾9 mm); a substantial loss of sensitivity was evident (83.33, 75.00 or 66.67%, respectively). At ROC analysis, the area under the curve was 0.8217 (s.e.=0.0573, 95% CI=0.70930–0.93411). The ROC curve is shown in Figure 1

.

Table 3 shows the number of EC delayed diagnoses and of unnecessary assessments that would occur by using different indicators to prompt immediate invasive diagnostic assessment: (a) different thickness cutoff, (b) presence of AUB, (c) suspicious TVUS findings or (d) different combinations of indicators (a+b, a+c). Endometrial thickness ⩾3 mm allowed 100% sensitivity, but would spare only 25.8% of invasive assessments. A higher proportion of invasive assessments was spared (44.5, 62.1, 76.1, 82.5%) by raising the thickness cutoff (⩾4, ⩾5 ⩾6, ⩾7 mm), although with a substantial loss in sensitivity. The presence of AUB was a rather specific (88.9%) but poorly sensitive indicator (41.6%), whereas the reverse was true for suspicious TVUS (757 negative or benign cases, 448 suspicious: sensitivity 81.8, specificity 63.2%). The combination of the presence of AUB and of a thickness ⩾4 mm allowed for a very favourable balance (sensitivity 91.6%, specificity 40.4%): raising the thickness cutoff to ⩾5 mm or ⩾6 mm further reduced unnecessary assessments (56.7 or 69.9%) with no loss in sensitivity.

Multivariate analysis of the association of age (continuous variable), endometrial thickness (continuous variable) and symptoms (present/absent) to EC occurrence within 2 years after TVUS is shown in Table 4

**Table 4:** Multivariate analysis of the association (RR=relative risk) of age (continuous variable), endometrial thickness (continuous variable) and symptoms (present/absent) to EC occurrence within 2 years from TVUS

**Variable**	**RR**	**95% CI**	**s.e.**	***P*-value**
Age	0.99	0.94–1.06	0.03	0.986
Thickness	1.15	1.03–1.29	006	0.010
Symptoms	6.23	1.91–20.30	3.75	0.002

. Age showed no significant association with EC risk. The presence of symptoms was associated with a significant independent increase of risk (6.23 : 1) as compared to asymptomatic status. Thickness showed also a significant association with EC risk increasing by 16% for each extra millimetre of thickness. When entered in the model, TVUS diagnosis (negative/benign *vs* suspicious of cancer) showed a nearly significant association with EC (RR=4.33, 95% CI 0.76–24.45), and symptoms remained the most significantly associated variable (RR=4.51; 95% CI 1.25–15.01) whereas thickness reduced its association, with risk increasing by 8% per millimetre increase, but at a nonsignificant level.

## DISCUSSION

The present study is based on a relatively large sample of breast cancer patients under tamoxifen treatment, followed up by cancer registry, and allows reliable considerations on the possible role of TVUS-assessed endometrial thickness as an indicator for invasive assessment aimed at early detection of EC.

This study confirms an increased risk of EC in subjects undergoing tamoxifen treatment, already reported in the literature (Fornander *et al*, 1989; Fisher *et al*, 1994), and in a previous study on a smaller cohort (Cecchini *et al*, 1998). If a selection bias is most likely in subjects self-referring for AUB, this is probably not true for asymptomatic subjects who were referred for TVUS as a routine follow-up procedure: although cases with AUB, which account for the majority of incident EC, were excluded from this subgroup, it still showed a significantly increased EC relative risk, exceeding four-folds that of the general population.

Endometrial thickness assessed at TVUS is commonly used as an indicator of EC risk (Karlsson *et al*, 1995; Weber *et al*, 1997). The decision of measuring single endometrial layer maximum thickness (rather than endometrium as a whole) was aimed at a better definition of cases with focal thickening, involving one single layer, which might be missed or underestimated at whole endometrium measurement; however, these cases are relatively rare, and in the large majority endometrial thickening is symmetrical and single-layer maximum thickness equals half the whole endometrium thickness.

The main problem with endometrial thickness in patients under tamoxifen treatment is that tamoxifen has been shown to induce subendometrial changes that simulate endometrial hyperplasia at TVUS (Goldstein, 1994), while atrophia is often the only finding at invasive assessment (Cecchini *et al*, 1998). Thus, the use of endometrial thickness as the only indicator for invasive assessment implies a large number of false positives, even at TVUS diagnosis, and of unnecessary assessments (Osmers *et al*, 1990; Ciatto *et al*, 1995).

In the present study only a cutoff of ⩾3 mm would be totally safe, granting for no missed cancer, but it would allow a limited (25.8%) reduction of unnecessary invasive assessments. Attempts to increase specificity and the proportion of spared unnecessary assessments by raising the ⩾3 mm cutoff led to a decrease of sensitivity, which became rapidly clinically unacceptable.

Combination with other clinical indicators such as the presence of AUB allowed to increase the thickness cutoff while maintaining good levels of sensitivity with a reduction of unnecessary assessment which is of clinical interest (AUB + thickness ⩾3, ⩾4 or ⩾5; sens.=100, 91.6 or 91.6%; spec.=22.8, 40.4 or 56.7%).

AUB is often considered sufficient to indicate invasive assessment in these patients, or even in the general population (Karlsson *et al*, 1995; Weber *et al*, 1997), but AUB is often spurious and poorly predictive of EC (the positive predictive value was only 3.65% in the present series). Abnormal uterine bleeding might not prompt invasive assessment when associated with low endometrial thickness: in the present study, 49 of 137 (35.77%) subjects with AUB had a thickness ⩽3 mm and showed no cancer at follow-up. These subjects might be better put under watchful waiting, in that spurious AUB is often anecdotal, whereas EC-related bleeding tends to persist. Our findings suggest adopting a cutoff that is substantially higher, and much rewarding in terms of spared unnecessary invasive assessments, as compared to a large study suggesting a cutoff of 2.5 mm (Smith-Bindman *et al*, 1998).

Periodic follow-up of breast cancer patients under tamoxifen treatment aimed at early detection of EC is a common practice, justified by the evidence of tamoxifen-induced increased risk of EC in these subjects. Although early detection of EC is possible by adopting intensive surveillance policies, there is no evidence supporting that early detection and treatment will improve EC prognosis, which is relatively good even in symptomatic cases, showing a very high cure rate. Although the difference is not statistically significant, it is worth noting that adverse EC prognostic indicators such as high histological grade (G3) and extensive (>50%) myometrial invasion were observed only in subjects with AUB at diagnosis: this might suggest delayed detection being associated with AUB, but this finding is based on a few cases and needs to be confirmed on a larger EC series. On the contrary, awareness of increased EC risk prompting special surveillance will cause further anxiety in these cancer patients, and false-positive findings, which may be relatively common, will cause unnecessary invasive assessments. A good specificity of the adopted surveillance protocol must thus be granted, and TVUS-assessed endometrial thickness seems to be a good indicator to be used, together with TVUS diagnostic findings and the presence of AUB, as a determinant for invasive assessment.
